# Ambulatory Health Care Visits Among Active Component Members of the U.S. Armed Forces, 2024

**Published:** 2025-09-20

This report documents the frequencies, rates, trends, and characteristics of ambulatory health care visits in 2024 of active component members of the U.S. Army, Navy, Air Force, Marine Corps, and Space Force. Ambulatory visits of U.S. service members in fixed military and non-military (reimbursed through the Military Health System) hospitals and clinics are documented by standardized records that are routinely archived in the Defense Medical Surveillance System (DMSS) for health surveillance purposes. Ambulatory visits not routinely and completely documented within fixed military and non-military hospitals and clinics (e.g., during deployments, field training exercises, or at sea) are not included in this analysis.

As in prior *MSMR* reports, all records of ambulatory health care visits by active component service members (ACSMs) were categorized according to the International Classification of Diseases, 10th Revision (ICD-10) codes entered in the primary (i.e., first-listed) diagnostic position of the visit records. Incidence rates were calculated per 1,000 person-years (p-yrs). Percent change in incidence was calculated using unrounded rates.

## Frequencies, rates and trends

**FIGURE 1.**
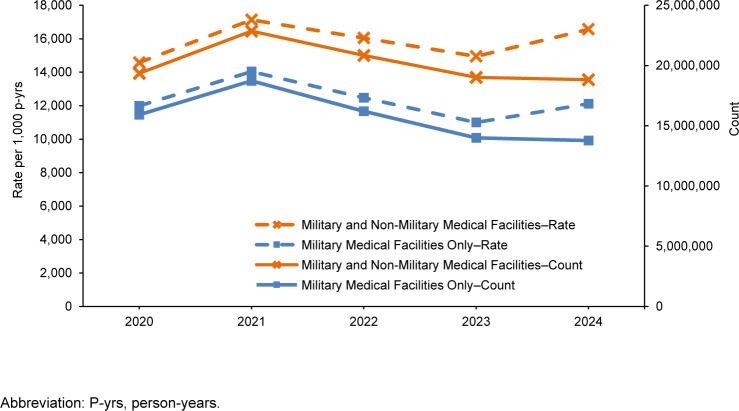
Counts and Rates of Ambulatory Visits by Year, Active Component, U.S. Armed Forces, 2020–2024

In 2024, U.S. ACSMs completed 18,821,239 ambulatory visits for medical care, resulting in a crude annual rate—for all causes—of 16,563.4 visits per 1,000 p-yrs, or 16.5 visits per p-yr **(Table 1)** . The observed rate represents an increase of 10.8% from 2023, despite the absolute number of visits continuing a decline from a peak in 2021 **(Figure 1)** . Excluding the ‘other’ major diagnostic category, there were 15,220.739 documented ambulatory visits for illnesses and injuries (ICD-10: A00–T88, including relevant pregnancy ‘Z’ codes) in 2024, corresponding to a crude rate of 13.4 visits per p-yr, which is 17.4% higher than in 2022 (11.4 per p-yr) and 55.6% higher than in 2020 (8.6 per p-yr).

**TABLE 1.** Numbers, Rates ^a^ and Ranks ^b^ of Ambulatory Visits, by ICD-10 Major Diagnostic Category, Active Component, U.S. Armed Forces, 2020, 2022 and 2024

	2020			2022			2024		
ICD-10 Major Diagnostic Category	No.	Rate	Rank	No.	Rate	Rank	No.	Rate	Rank
Musculoskeletal system (M00–M99)	3,617,917	2,726.3	2	5,008,322	3,857.1	2	5,293,151	4,658.2	1
Other (Z00–Z99, except pregnancy-related) ^c^	7,919,267	5,967.6	1	6,024,545	4,639.7	1	3,600,500	3,168.6	2
Mental disorders (F01–F99)	2,087,067	1,572.7	3	2,649,250	2,040.3	3	2,737,955	2,409.5	3
Nervous system and sensory organ disorders (G00–G99, H00–H95)	1,331,414	1,003.3	4	1,698,433	1,308.0	4	1,843,803	1,622.6	4
Signs, symptoms, ill-defined conditions (R00–R99)	1,233,754	929.7	5	1,557,645	1,199.6	5	1,487,884	1,309.4	5
Injury, poisoning (S00–T88, DOD0101–DOD0105)	679,734	512.2	6	862,796	664.5	6	866,110	762.2	6
Respiratory system (J00–J99, U07.0)	511,242	385.2	7	581,557	447.9	7	652,743	574.4	7
Skin and subcutaneous tissue diseases (L00–L99)	348,221	262.4	9	434,957	335.0	8	432,238	380.4	8
Pregnancy and delivery (O00–O9A, relevant Z codes)	365,569	275.5	8	414,311	319.1	9	400,117	352.1	9
Pregnancy and delivery, females only (O00–O9A, relevant Z codes)		1,604.6			1,825.7			1,971.1	
Genitourinary system (N00–N99)	286,276	215.7	10	344,783	265.5	10	354,320	311.8	10
Digestive system (K00–K95)	228,903	172.5	11	299,015	230.3	11	326,319	287.2	11
Endocrine, nutritional, metabolic diseases (E00–E89)	138,273	104.2	13	177,188	136.5	13	213,415	187.8	12
Infectious and parasitic diseases (A00–B99)	211,744	159.6	12	214,398	165.1	12	190,315	167.5	13
Circulatory system (I00–I99)	129112	97.3	14	170667	131.4	15	170386	149.9	14
Neoplasms (C00–D49)	115,404	87.0	15	146,859	113.1	16	146,410	128.8	15
Hematological and immune disorders (D50–D89)	37,967	28.6	17	48,612	37.4	17	49,258	43.3	16
Congenital anomalies (Q00–Q99)	18,275	13.8	18	28,917	22.3	18	29,395	25.9	17
COVID-19 (U07.1, U09.9)	84,793	63.9	16	172,606	132.9	14	26,920	23.7	18
Total	19,344,932	14,577.4		20,834,861	16,045.7		18,821,239	16,563.4	

Abbreviations: ICD, International Classification of Diseases; No., number.

a Rate per 1,000 person-years.

b Rank of major diagnostic category based on number of ambulatory visits.

c Other factors influencing health status and contact with health services (excluding pregnancy-related).

A ‘Z’ code in the first diagnostic position identifies administrative visits within the ‘other’ category that reflects the care related to other factors influencing health status and contact with health services (excluding pregnancy). After a sharp decline observed in Z-coded encounters in 2023 ^1^ compared to 2021 and 2019, the frequency in 2024 remained relatively stable, with a slight 3.7% increase over the previous year (data not shown).

## Ambulatory visits, by ICD-10 major diagnostic categories

As in prior years, the leading 5 major diagnostic categories in 2024 remained consistent, accounting for almost four-fifths (79.5%) of all ambulatory visits among ACSMs. Musculoskeletal system/connective tissue disorders (28.1%) rose to become the leading category in 2024, surpassing ‘other’ (19.1%), which was the dominant category in 2020 and 2022. Mental health disorders (14.5%), disorders of the nervous system and sensory organs (9.8%), and signs, symptoms and ill-defined conditions (7.9%) maintained stable rankings **(Table 1)** . Rankings for other diagnostic categories were largely stable, with only a modest shift in endocrine disorders surpassing infectious diseases. In contrast, COVID-19 fell to the lowest rank, representing only 0.1% of visits, down from 0.8% in 2022, reflecting pandemic peak and decline.

**FIGURE 2.**
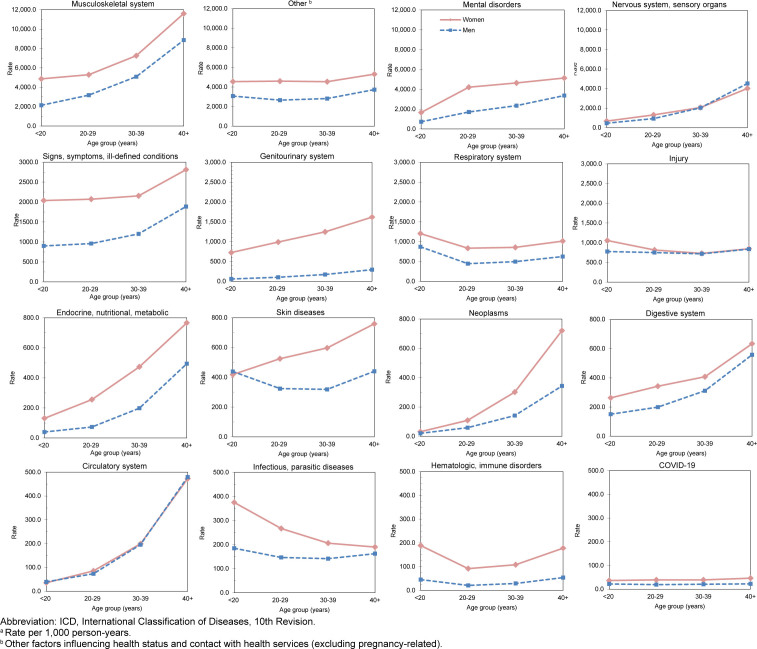
Rates ^a^ of Ambulatory Visits by ICD-10 Major Diagnostic Category, Age Group, and Sex, Active component, U.S. Armed Forces, 2024

Excluding the ‘other’ category, rates of ambulatory visits increased in all but 1 of the 17 major diagnostic categories of illnesses and injuries between 2020 and 2024. As in prior years, diagnostic ‘S’ codes (for injuries), as opposed to ‘T’ codes (burns and poisonings), accounted for nearly 90% of all ambulatory encounters within this major diagnostic category (data not shown). Excluding the ‘other’ major diagnostic category, COVID-19 was the sole diagnostic category to decline in both numbers and rates within the illness and injury major category, with visits decreasing by 62.9%. Musculoskeletal system conditions accounted for the highest growth in ambulatory visits, totaling an additional 1,675,234 visits (rate increase of 70.9%) from 2020 to 2024, followed by mental health disorders (650,888 more visits, 53.2% rate increase). Except for infectious diseases and pregnancy and delivery-related visits, all other diagnostic categories exhibited rate increases exceeding 40% **(Figure 2)** . Infectious diseases increased by only 5%, while pregnancy and delivery-related visits increased by 27.8%.

**What are the new findings?**

In 2024 the rate of ambulatory visits in U.S. military and non-military medical facilities was 16.5 visits per person-year, 10.8% higher than the 2023 rate. Excluding administrative visits, the crude annual rate of 13.4 visits per person-year for illnesses and injuries in 2024 was 17.4% higher than in 2022 and 55.6% higher than in 2020. The numbers and rates of primary causes for ambulatory visits have increased in 16 of 18 diagnostic categories from 2020 to 2024, except for ‘other’ and COVID-19 diagnoses. Musculoskeletal, mental, and nervous system or sensory organ disorders remain the leading causes of ambulatory visits, with substantial increases from 2020 to 2024. Musculoskeletal disorders showed the largest absolute ambulatory visit increase, with 1,675,234 total additional visits in 2024 in comparison to 2020, followed by mental health disorders, which increased by 650,888 visits during the same period.

**What is the impact on readiness and force health protection?**

Disorders of the musculoskeletal, mental, and nervous system and sensory organ major diagnostic categories are already known to have significant impacts on the well-being of military personnel and operational readiness. Unaddressed musculoskeletal injuries and mental health disorders may lead to prolonged periods of unoccupied time, reduced ability to meet the physical and psychological demands of military service, and contribute to attrition.

## Ambulatory visits, by sex

For both male and female ACSMs, joint pain comprised over 40% of all diagnoses within the musculoskeletal system category. Adjustment disorder was the leading diagnosis in the mental health category, representing approximately 20% among both sexes **(Tables 2** and **3)** . Unspecified and iron deficiency types of anemia were among the leading diagnoses within the hematological and immune disorders major diagnostic category, accounting for 28.2% and 56.7% of diagnoses among service men and women, respectively **(Tables 2** and **3)** . Unspecified viral infection and unspecified acute upper respiratory infection were the leading diagnoses in 2024 for infectious and parasitic diseases and disorders of the respiratory system, respectively **(Tables 2** and **3)** . While congenital anomalies were not frequently diagnosed among women, nearly a quarter (24.4%) of the congenital anomalies in men were attributed to congenital deformities of feet, including congenital *pes planus* (flat foot) and congenital *pes cavus* (high arch) **(Table 2)** .

**TABLE 2.** Numbers and Percentages of the Most Frequent Diagnoses During Ambulatory Visits Among Men ♂, by ICD-10 Major Diagnostic Category, Active Component, U.S. Armed Forces, 2024

Diagnostic Category (ICD-10 codes)	No.	% ^a^
**Infectious and parasitic diseases (A00–B99)**	**139,390**	
Viral infection, unspecified	23,121	16.6
Viral wart, unspecified	8,825	6.3
Tinea unguium	8,411	6.0
Viral intestinal infection, unspecified	8,198	5.9
Plantar wart	6,835	4.9
**Neoplasms (C00–D49)**	**103,508**	
Neoplasm of uncertain behavior of skin	10,423	10.1
Melanocytic nevi, unspecified	7,367	7.1
Melanocytic nevi of trunk	4,132	4.0
Benign lipomatous neoplasm, unspecified	3,832	3.7
Malignant neoplasm of testis, unspecified whether descended or undescended	3,313	3.2
**Endocrine, nutritional, metabolic diseases (E00–E89)**	**141,812**	
Obesity, unspecified	19,474	13.7
Hyperlipidemia, unspecified	17,090	12.1
Testicular hypofunction	14,072	9.9
Vitamin D deficiency, unspecified	13,354	9.4
Type 2 diabetes mellitus without complications	11,147	7.9
**Hematological and immune disorders (D50–D89)**	**26,681**	
Anemia, unspecified	5,122	19.2
Other specified disorders of white blood cells	2,945	11.0
Sickle cell trait	2,440	9.1
Iron deficiency anemia, unspecified	2,405	9.0
Glucose-6-phosphate dehydrogenase (G6PD)	1,691	6.3
**Mental disorders (F01–F99)**	**1,880,354**	
Adjustment disorder	354,778	18.9
Alcohol dependence	242,595	12.9
Post-traumatic stress disorder (PTSD)	240,217	12.8
Anxiety disorder, unspecified	130,854	7.0
Generalized anxiety disorder	115,299	6.1
**Nervous system and sensory organ disorders (G00–G99, H00–H95)**	**1,496,485**	
Sleep apnea	659,207	44.1
Myopia	113,240	7.6
Chronic pain, not elsewhere classified	61,735	4.1
Insomnia	44,903	3.0
Astigmatism	40,782	2.7
**Circulatory system (I00–I99)**	**140,343**	
Essential (primary) hypertension	65,148	46.4
Scrotal varices	4,490	3.2
Atherosclerotic heart disease of native coronary artery	3,377	2.4
Pulmonary embolism without acute cor pulmonale	2,920	2.1
Acute embolism, thrombosis of deep veins of lower extremity	2,881	2.1
**Respiratory system (J00–J99)**	**473,115**	
Acute upper respiratory infection, unspecified	130,351	27.6
Acute pharyngitis, unspecified	45,874	9.7
Allergic rhinitis due to pollen	35,517	7.5
Allergic rhinitis, unspecified	24,264	5.1
Pneumonia, unspecified organism	23,712	5.0
**Digestive system (K00–K95)**	**249,155**	
Gastroesophageal reflux disease without esophagitis	32,509	13.0
Non-infective gastroenteritis, colitis, unspecified	25,322	10.2
Melena	10,585	4.2
Constipation	10,179	4.1
Hemorrhage of anus and rectum	9,037	3.6
**Genitourinary system (N00–N99)**	**131,311**	
Other specified disorders of male genital organs	26,062	19.8
Male erectile dysfunction, unspecified	15,795	12.0
Calculus of kidney	8,862	6.7
Hypertrophy of breast	6,394	4.9
Male infertility, unspecified	5,208	4.0
**Skin and subcutaneous tissue (L00–L99)**	**319,032**	
Pseudofolliculitis barbae	46,211	14.5
Acne vulgaris	24,635	7.7
Dermatitis, unspecified	21,560	6.8
Ingrowing nail	17,487	5.5
Pilonidal cyst and sinus without abscess	9,979	3.1
**Musculoskeletal system, connective tissue (M00–M99)**	**3,999,023**	
Pain in joint	1,688,914	42.2
Low back pain	592,419	14.8
Pain in limb, hand, foot, fingers, toes	319,851	8.0
Cervicalgia	184,082	4.6
Dorsalgia, unspecified	104,525	2.6
**Congenital anomalies (Q00–Q99)**	**20,955**	
Other specified congenital malformations of skin	2,650	12.6
Congenital *pes planus*	2,516	12.0
Congenital *pes cavus*	1,624	7.7
Other congenital deformities of feet	989	4.7
Atrial septal defect	903	4.3
**Symptoms, signs, abnormal clinical and laboratory findings, not elsewhere classified (R00–R99)**	**1,049,838**	
Other symptoms and signs involving emotional state	74,864	7.1
Headache, unspecified	56,031	5.3
Chest pain, unspecified	51,606	4.9
Other abnormalities of breathing	39,718	3.8
Unspecified abdominal pain	38,734	3.7
**Injury, poisoning (S00–T98, DOD0101–DOD0105)**	**701,386**	
Sprain of ankle	41,361	5.9
Concussion	27,000	3.8
Sprain of shoulder joint	25,685	3.7
Sprain of cruciate ligament of knee	25,065	3.6
Unspecified injury of ankle, foot	18,085	2.6
** Other (Z00–Z99, except pregnancy-related) ^b^**	**2,658,328**	
Encounter for other specified special examination	214,061	8.1
Encounter for administrative examinations, unspecified	207,888	7.8
Encounter for immunization	192,791	7.3
Encounter for issue of medical certificate	181,653	6.8
Encounter for other administrative examination	142,319	5.4

Abbreviations: ICD, International Classification of Diseases, 10th Revision; No., number; G6PD, glucose-6-phosphate dehydrogenase.

a Percentage of the total number of hospitalizations within the diagnostic category.

b Other factors influencing health status and contact with health services (excluding pregnancy-related).

**TABLE 3.** Numbers and Percentages of the Most Frequent Diagnoses During Ambulatory Visits Among Women ♀, by ICD-10 Major Diagnostic Category, Active Component, U.S. Armed Forces, 2024

Diagnostic Category (ICD-10 codes)	No.	% ^a^
**Infectious and parasitic diseases (A00–B99)**	**50,925**	
Viral infection, unspecified	9,377	18.4
Candidiasis of vulva and vagina	5,093	10.0
Viral intestinal infection, unspecified	2,791	5.5
Other viral agents as the cause of disease classified elsewhere	2,683	5.3
Tinea unguium	1,919	3.8
**Neoplasms (C00–D49)**	**42,902**	
Leiomyoma of uterus, unspecified	5,567	13.0
Malignant neoplasm of breast of unspecified site	3,631	8.5
Neoplasm of uncertain behavior of skin	3,079	7.2
Melanocytic nevi, unspecified	2,625	6.1
Benign neoplasm of pituitary gland	1,432	3.3
**Endocrine, nutritional, metabolic diseases (E00–E89)**	**71,603**	
Obesity, unspecified	13,890	19.4
Vitamin D deficiency, unspecified	7,996	11.2
Polycystic ovarian syndrome	7,116	9.9
Hypothyroidism, unspecified	5,791	8.1
Overweight	4,102	5.7
**Hematological and immune disorders (D50–D89)**	**22,577**	
Iron deficiency anemia, unspecified	6,476	28.7
Anemia, unspecified	6,312	28.0
Sickle cell trait	1,225	5.4
Iron deficiency anemia secondary to blood loss (chronic)	1,139	5.0
Other specified disorders of white blood cells	1,103	4.9
**Mental disorders (F01–F99)**	**857,601**	
Adjustment disorder	176,933	20.6
Post-traumatic stress disorder (PTSD)	129,684	15.1
Generalized anxiety disorder	83,008	9.7
Anxiety disorder, unspecified	72,758	8.5
Major depressive disorder, recurrent / moderate	49,304	5.7
**Nervous system and sensory organ disorders (G00–G99, H00–H95)**	**347,318**	
Sleep apnea	59,172	17.0
Myopia	41,002	11.8
Chronic pain, not elsewhere classified	21,840	6.3
Migraine, unspecified	19,925	5.7
Insomnia 13,558	3.9	
**Circulatory system (I00–I99)**	**30,043**	
Essential (primary) hypertension	10,776	35.9
Supraventricular tachycardia	1,164	3.9
Varicose veins of lower extremities with other complications	1,103	3.7
Venous insufficiency (chronic) (peripheral)	892	3.0
Raynaud's syndrome	810	2.7
**Respiratory system (J00–J99)**	**179,628**	
Acute upper respiratory infection, unspecified	51,783	28.8
Acute pharyngitis, unspecified	20,994	11.7
Allergic rhinitis due to pollen	13,582	7.6
Allergic rhinitis, unspecified	9,943	5.5
Acute nasopharyngitis [common cold]	8,022	4.5
**Digestive system (K00–K95)**	**77,164**	
Constipation	11,880	15.4
Non-infective gastroenteritis and colitis, unspecified	8,700	11.3
Gastroesophageal reflux disease without esophagitis	8,193	10.6
Unspecified hemorrhoids	2,541	3.3
Melena	2,271	2.9
**Genitourinary system (N00–N99)**	**223,009**	
Acute vaginitis	17,376	7.8
Stress incontinence, female / male	17,255	7.7
Urinary tract infection, site not specified	16,538	7.4
Abnormal uterine and vaginal bleeding, unspecified	15,477	6.9
Other specified non-inflammatory disorders of vagina	14,624	6.6
**Pregnancy and childbirth (O00–O99, relevant Z codes)**	**400,117**	
Encounter for care and examination of lactating mother	57,402	14.3
Pregnant state, incidental	32,117	8.0
Encounter for supervision of normal first pregnancy	18,316	4.6
Encounter for supervision of other normal pregnancy	17,383	4.3
Other specified pregnancy-related conditions	15,244	3.8
**Skin and subcutaneous tissue (L00–L99)**	**113,206**	
Acne vulgaris	19,533	17.3
Dermatitis, unspecified	8,699	7.7
Urticaria, unspecified	3,897	3.4
Non-scarring hair loss, unspecified	3,562	3.1
Ingrowing nail	3,320	2.9
**Musculoskeletal system and connective tissue (M00–M99)**	**1,294,128**	
Pain in joint	537,864	41.6
Low back pain	182,116	14.1
Pain in limb, hand, foot, fingers, toes	107,462	8.3
Cervicalgia	70,329	5.4
Dorsalgia, unspecified	35,506	2.7
**Symptoms, signs, abnormal clinical and laboratory findings, not elsewhere classified (R00–R99)**	**438,046**	
Pelvic, perineal pain	30,466	7.0
Headache, unspecified	27,539	6.3
Unspecified abdominal pain	25,013	5.7
Other symptoms and signs involving emotional state	24,597	5.6
Chest pain, unspecified	14,445	3.3
**Injury, poisoning (S00–T98, DOD0101–DOD0105)**	**164,724**	
Sprain of ankle	13,372	8.1
Concussion	7,464	4.5
Sprain of cruciate ligament of knee	5,661	3.4
Unspecified injury of ankle, foot	4,641	2.8
Injury of muscle, fascia, tendon of abdomen, lower back, pelvis	3,767	2.3
** Other (Z00–Z99, except pregnancy-related) ^b^**	**942,172**	
Encounter for administrative examinations, unspecified	59,790	6.3
Encounter for immunization	49,780	5.3
Encounter for other specified special examinations	46,775	5.0
Encounter for issue of medical certificate	46,351	4.9
Other specified counseling	43,523	4.6

Abbreviations: ICD, International Classification of Diseases, 10th Revision; No., number.

a Percentage of the total number of hospitalizations within the diagnostic category.

b Other factors influencing health status and contact with health services (excluding pregnancy-related).

In 2024, service men accounted for nearly three-fourths (71.6%) of all illness- and injury-related visits, but the annual crude rate for service women (21.3 visits per p-yr) was 82.8% higher than among service men (11.7 visits per p-yr) (data not shown). Excluding pregnancy- and delivery-related visits, which accounted for 9.4% of all non-Z-coded ambulatory visits among service women, the illness and injury ambulatory visit rate was 19.4 visits per p-yr, 65.9% higher than among service men.

Rates of illness- and injury-specific diagnoses among service women exceeded male rates by 50% in all major diagnostic categories except diagnoses for nervous system and sensory organs, circulatory system, digestive system, and injury (data not shown). Female rates were more than twice those of male rates for conditions in the hematological, mental, genitourinary, and endocrine-, nutritional- and metabolic-related disorder categories.

Relationships between age group and ambulatory visit rates were broadly similar among men and women within all diagnostic categories **(Figure 2)** . Ambulatory rates for musculoskeletal system, mental health disorders, neoplasms, disorders in nervous, digestive, circulatory systems, and endocrine-, nutritional- and metabolic-related conditions rose more steeply with advancing age than other categories of illness or injury **(Figure 2)** .

**FIGURE 3.**
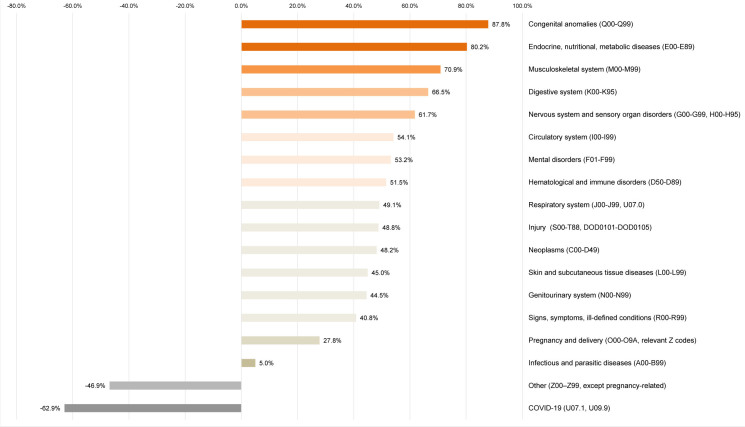
Rate Changes in Ambulatory Visits by ICD-10 Major Diagnostic Category, U.S. Armed Forces, 2020–2024

Eight of the 10 leading diagnoses among ambulatory visits were the same for male and female service members: pain in joint; lower back pain; adjustment disorders; pain in limb, hand, foot, fingers, or toes; post-traumatic stress disorder (PTSD); cervicalgia (neck pain); unspecified anxiety disorder; and sleep apnea. Sleep apnea was the second-most frequent illness- or injury-specific primary diagnosis for men, but ninth for women. The difference in the rate rank order of mental disorders is also worth noting. Alcohol dependence and unspecified acute respiratory infections were the sixth and tenth most frequent diagnoses, respectively, for men but were not identified among the leading 10 causes of ambulatory visits for women, while generalized anxiety disorder and unspecified dorsalgia were among the 10 most common diagnoses for women **(Tables 2** and **3)** .

## Discussion

In 2024, ambulatory visits among ACSMs increased by 10.8% compared to 2023, reaching 16.5 visits per person-year, although remaining below the 2021 peak. Rates for all major diagnostic categories increased, with the exception of the ‘other’ major diagnostic category and COVID-19. The largest absolute increase in the number of ambulatory visits was observed for musculoskeletal system disorders, which surpassed the ‘other’ category as the most frequent diagnosis. Notable growth was also seen within the mental health, nervous system, injury, and respiratory system categories. While infectious, endocrine, circulatory, neoplasms, hematological, and congenital anomalies experienced modest increases, their rankings remained relatively stable, at the lower end of the spectrum. When excluding visits documented by ICD-10 Z codes, the rate of illness- and injury-specific ambulatory visits was approximately 17% higher than in 2022, and over 55% higher than in 2020. The rate of encounters for COVID-19 peaked in 2022 (when it ranked fourteenth) and then sharply declined to last place by 2024, reflecting the pandemic peak and decline.

The sex-specific rate ratio for illness and injury-specific ambulatory encounters showed that female service members used outpatient care more often than their male counterparts (21.3 vs. 11.7 visits per p-yr, respectively). This is consistent with a recent report based on the 2022 National Ambulatory Medical Care Survey indicating that civilian women use health care services approximately 1.8 times more than civilian men. ^2^ The crude annual rate of illness- and injury-related visits among ACSMs (13.4 visits per p-yr), however, far exceeds the rate of ambulatory visits among civilians ages 18-44 years (324.6 visits per 1,000 persons, or about 0.3 visits per p-yr). ^2^ Future analyses comparing the major diagnostic category rates to civilian counterparts may be useful to further elucidate the costs of readiness.

Several limitations should be considered when interpreting these findings. Unit level ambulatory care, care by non-credentialed providers (e.g., medics, corpsmen), and at deployed medical treatment facilities (including ships at sea) are not included. This summary does not reflect that the nature and rates of illnesses and injuries may vary between deployed and non-deployed ACSMs.

Prior reports described the number of virtual versus in-person ambulatory encounters, but data quality issues about the variable delineating this encounter type have also been identified; it is an area of active inquiry.

This summary is based on primary (i.e., first-listed) diagnosis codes reported on ambulatory visit records, and the current summary discounts morbidity related to co-morbid and complicating conditions that may have been documented in secondary diagnostic positions within health care records. The accuracy of reported diagnoses likely varies according to medical condition, clinical setting, care provider, and treatment facility, as the information is collected for non-surveillance purposes. Although specific diagnoses during individual encounters were potentially not definitive, final, or even correct, summaries of the frequencies, trends, and natures of ambulatory encounters among ACSMs provide descriptive evidence to inform further research and evaluation.

Rates and frequencies reported do not reflect unique individuals, but a rate of total ambulatory visits per person-year. This report documents all ambulatory health care visits but does not estimate incidence rates for the diagnoses described. These data provide descriptors for health care provision, which elevate rates for disorders requiring increased numbers of ambulatory visits. In contrast to common, self-limited, and minor illnesses and injuries that require little, if any, follow-up or continuing care, illnesses and injuries necessitating multiple ambulatory visits for evaluation, treatment, and rehabilitation are over-represented in this summary.
